# Epidemiology and treatment outcomes of TB–HIV co-infection in a rural South African setting: an exploratory analysis of retreatment and contextual factors

**DOI:** 10.3389/fpubh.2026.1792518

**Published:** 2026-06-26

**Authors:** Luzuko Mkono, Ncomeka Sineke, Ntandazo Dlatu, Mojisola Clara Hosu, Teke Apalata, Lindiwe M. Faye

**Affiliations:** 1 Division of Medical Microbiology, Department of Laboratory-Medicine and Pathology, Faculty of Health Sciences, Walter Sisulu University, Mthatha, Eastern Cape, South Africa; 2 School of Public Health, Faculty of Medicine and Health Sciences, Walter Sisulu Institute for Clinical Governance, Healthcare Administration, Walter Sisulu University, Mthatha, South Africa

**Keywords:** epidemiology, HIV, logistic regression, machine learning, random forest, rural South Africa, TB–HIV co-infection, treatment outcomes

## Abstract

**Background:**

Tuberculosis–HIV (TB–HIV) co-infection remains a major public health challenge in South Africa, particularly in rural settings where clinical, socioeconomic, and health-system factors may influence treatment outcomes. This study examined the epidemiological characteristics of TB–HIV co-infection and factors associated with tuberculosis treatment outcomes in a rural Eastern Cape cohort.

**Methods:**

A retrospective cohort study was conducted using routinely collected programmatic data from 422 adult tuberculosis patients treated between 2018 and 2022. Baseline demographic and clinical characteristics were summarized descriptively. Associations between patient characteristics and treatment success were evaluated using univariable and multivariable logistic regression analyses. An exploratory Random Forest (RF) model was additionally applied to assess predictive performance and identify variables contributing to outcome classification. Model performance was evaluated using Precision–Recall curves and Average Precision (AP).

**Results:**

The prevalence of TB–HIV co-infection was 57.8%. Treatment success rates were similar between TB–HIV co-infected and TB-only patients, and HIV status was not independently associated with treatment success in adjusted analyses. Previous TB treatment was associated with lower treatment success in univariable analysis (OR = 0.48; 95% CI: 0.29–0.78), although this association did not remain statistically significant after adjustment. Mortality was 6.6% among TB–HIV co-infected patients and 10.7% among TB-only patients, with no statistically significant difference between groups. The RF model demonstrated higher predictive performance (AP = 0.907) than logistic regression (AP = 0.810). Feature importance analysis identified socioeconomic characteristics, including education, income source, and employment status, as important contributors to model performance.

**Conclusion:**

TB–HIV co-infection was highly prevalent in this rural cohort; however, treatment success and mortality outcomes were comparable between TB–HIV co-infected and TB-only patients. While HIV status was not independently associated with treatment outcomes, the absence of detailed HIV-related clinical information limits the interpretation of the underlying mechanisms. The exploratory machine-learning analysis suggests that socioeconomic factors may contribute to outcome prediction, highlighting the importance of considering broader contextual influences alongside clinical characteristics when designing interventions to improve tuberculosis treatment outcomes.

## 1 Introduction

Tuberculosis (TB), caused by *Mycobacterium tuberculosis*, remains one of the leading infectious causes of morbidity and mortality worldwide. An estimated one-quarter of the global population is infected with *M. tuberculosis*, and approximately 10 million people develop active TB each year (1, 2). Despite substantial advances in diagnosis, treatment, and prevention, TB continues to pose a major public health challenge, particularly in low- and middle-income countries. South Africa is among the 30 highest TB-burden countries globally, with incidence rates exceeding 500 cases per 100,000 population (3, 4). The burden of disease is especially pronounced in resource-limited rural provinces such as the Eastern Cape, where poverty, labor migration, geographic barriers to healthcare access, and health system constraints contribute to ongoing transmission and adverse health outcomes (5, 6).

The Human Immunodeficiency Virus (HIV) epidemic further amplifies the TB burden. HIV infection is the strongest known risk factor for progression from latent TB infection to active disease and is associated with increased susceptibility to recurrent TB, severe clinical presentation, and mortality (7, 8). South Africa continues to have one of the highest HIV prevalence rates globally, with substantial regional variation and persistently high rates in the Eastern Cape Province (9, 10). Consequently, TB–HIV co-infection remains a major public health concern, placing considerable demands on healthcare services and TB control programs.

To address this dual burden, integrated TB–HIV services have been widely implemented across South Africa to improve case detection, facilitate timely initiation of treatment, and strengthen continuity of care. Previous studies have reported improvements in survival and treatment outcomes following the scale-up of integrated services and antiretroviral therapy (ART) programs (11–13). However, treatment outcomes remain heterogeneous across settings, particularly in rural and underserved communities where socioeconomic disadvantage, barriers to healthcare access, and programmatic challenges may influence patient outcomes. Furthermore, while clinical factors are recognized determinants of treatment success, increasing evidence suggests that broader contextual influences, including education, employment, income security, and other social determinants of health, may also contribute to variations in treatment outcomes.

Understanding the factors associated with treatment outcomes among TB–HIV co-infected patients is particularly important in rural settings, where both HIV prevalence and structural vulnerabilities remain high. However, empirical evidence from rural Eastern Cape populations remains limited. In addition, routinely collected program data are often constrained by the absence of detailed clinical and health-system variables, creating uncertainty regarding the relative contributions of patient-level, contextual, and healthcare-related factors to treatment outcomes. Generating locally relevant evidence is therefore essential for informing program planning, identifying vulnerable populations, and strengthening tuberculosis care in high-burden settings.

Against this background, this study examined the epidemiology and treatment outcomes of tuberculosis patients with and without HIV co-infection in a rural Eastern Cape cohort. Specifically, the study aimed to: (i) describe the demographic, clinical, and treatment outcome characteristics of patients with and without HIV co-infection; (ii) assess factors associated with treatment success using conventional regression approaches; and (iii) explore the contribution of demographic, clinical, and socioeconomic variables to outcome prediction using a machine-learning framework. By combining epidemiological and exploratory predictive analyses, the study sought to provide insight into factors associated with tuberculosis treatment outcomes in a resource-limited rural setting.

## 2 Materials and methods

### 2.1 Study design and setting

A retrospective cohort study was conducted using routinely collected tuberculosis (TB) and human immunodeficiency virus (HIV) program data from public healthcare facilities in a rural district of the Eastern Cape Province, South Africa. The study utilized integrated TB and HIV program records to obtain demographic, clinical, and treatment outcome information for adult patients receiving TB treatment between 2018 and 2022. The Eastern Cape is among South Africa’s highest TB- and HIV-burden provinces and is characterized by substantial socioeconomic and healthcare challenges, particularly in rural communities. These challenges include high levels of poverty and unemployment, geographic barriers to healthcare access, and limitations in healthcare resources that may influence disease management and treatment outcomes. TB and HIV services are primarily delivered through an integrated network of primary healthcare clinics, community health centers, and district hospitals, supported by national TB control strategies, directly observed treatment (DOT) services, and HIV care programs. The use of routinely collected program data provides an opportunity to evaluate treatment outcomes under real-world service delivery conditions and to identify factors associated with treatment success within a high-burden rural setting. Given the substantial burden of TB–HIV co-infection in the Eastern Cape, understanding treatment outcomes in this context is important for informing program planning, resource allocation, and strategies aimed at improving patient care.

### 2.2 Study population

The study cohort comprised 422 adult patients (≥18 years) diagnosed with tuberculosis (TB) and initiated on anti-tuberculosis treatment between January 2018 and December 2022. Patients were identified from routinely collected TB and HIV program records maintained at participating healthcare facilities.

Eligible participants were required to have a documented HIV status and a recorded TB treatment outcome to allow comparative analyses of treatment outcomes between TB–HIV co-infected and TB-only patients. Treatment outcomes were classified according to national tuberculosis program definitions and included treatment success (cure or treatment completion), death, treatment failure, and loss to follow-up. Patients recorded as “transferred out,” “moved out,” or “still on treatment” at the time of data extraction were excluded from outcome analyses because their final treatment outcomes could not be reliably determined. These exclusions were applied to minimize outcome misclassification and ensure the accuracy of treatment outcome assessments. The final analytical cohort, therefore, consisted of patients with complete outcome information available within the program records.

### 2.3 Data quality and missing data

Data were extracted from routinely collected registers of the tuberculosis and HIV programs. Before analysis, records were screened for completeness, consistency, and eligibility. Variables were reviewed for missing values, implausible entries, and duplicate records. Patients without documented HIV status or treatment outcomes were excluded from the study because these variables were essential for the primary analyses. The extent of missingness for demographic, socioeconomic, and clinical variables was assessed descriptively. Analyses were conducted using a complete-case approach, in which only observations with complete information for the variables included in a specific model were analyzed. Multiple imputation was not performed because the proportion of missing data was low for the variables retained in the final models and because additional information was unavailable to reliably inform imputation. The extent of missingness and the final number of observations included in each analysis are reported in the Results section.

### 2.4 Study outcomes and predictors

The primary outcome was TB treatment success, defined as patients who were cured or completed treatment in accordance with national and World Health Organization guidelines. The secondary outcome was unsuccessful treatment, defined as treatment failure, loss to follow-up, or death during treatment.

Predictors included demographic and clinical variables routinely captured in program data:

- Demographic variables: age, sex, education level, income source, and employment status
- Clinical variables: HIV status, TB type (pulmonary/extrapulmonary), previous TB history (new/retreatment), drug-resistance profile, and comorbidities
- Behavioral variables: smoking and alcohol use
- Facility-level variables: district, facility location, and level of care

Socioeconomic variables (e.g., education, income, employment) were included as contextual indicators of patient-level conditions that may influence treatment adherence and outcomes. These variables were interpreted cautiously as indirect measures rather than direct representations of broader social or engagement constructs.

### 2.5 Descriptive and epidemiological analysis

Descriptive statistics were used to summarize baseline characteristics. Continuous variables were reported as medians and interquartile ranges (IQR), while categorical variables were presented as counts and percentages.

Comparisons between TB–HIV co-infected and TB-only patients were performed using the Mann–Whitney U test for continuous variables and Pearson’s chi-square test for categorical variables. Statistical significance was set at *p* < 0.05.

### 2.6 Multivariable analysis

Multivariable analyses were conducted to identify factors independently associated with tuberculosis treatment success, while controlling for potential confounders. A two-step modeling strategy was employed. First, univariable logistic regression analyses were performed to examine associations between individual predictors and treatment success. Variables with a *p*-value < 0.20, together with variables considered clinically relevant based on existing literature and program importance (including age, sex, HIV status, TB type, and previous TB treatment history), were considered for inclusion in multivariable models. Second, multivariable logistic regression was used to estimate adjusted odds ratios (AORs) and 95% confidence intervals (CIs). Given the relatively high treatment success rate in the cohort, Poisson regression with robust variance was also fitted to estimate adjusted risk ratios (aRRs), which provide more interpretable effect estimates when outcomes are common. Model adequacy was assessed using likelihood ratio tests and the Akaike Information Criterion (AIC). Results from both modeling approaches were compared to evaluate the consistency and robustness of the findings.

### 2.7 Exploratory machine learning analysis

As a supplementary analysis, a Random Forest classifier was developed to explore whether complex, non-linear relationships among demographic, clinical, and socioeconomic variables could provide additional insights into treatment outcomes. The analysis was intended to complement, rather than replace, the regression-based approaches. Given the modest sample size and observational design, the Random Forest analysis was undertaken as an exploratory, hypothesis-generating exercise. Model performance was assessed using cross-validation, and feature importance measures were used to identify variables contributing to classification performance. These findings were interpreted cautiously and were not considered evidence of causal or independent associations.

### 2.8 Model validation and interpretation

Model validation was performed using 5-fold stratified cross-validation to ensure consistent outcome distribution across training and test sets.

A decision tree was additionally generated to visualize hierarchical relationships between predictors and treatment outcomes. This provided a simplified representation of how key variables partitioned patients into different outcome groups.

### 2.9 Ethical considerations

This study was conducted in accordance with the Declaration of Helsinki. Approval was granted by the Research Ethics and Biosafety Committee of the Faculty of Health Sciences at Walter Sisulu University (Reference No. 140/2025, 02 July 2025) and the Eastern Cape Department of Health (Reference No. EC_202507_022; 11 July 2025).

## 3 Results

### 3.1 Data quality

A total of 422 patient records met the study inclusion criteria and were available for analysis. Records without documented HIV status or treatment outcomes were excluded during data cleaning. Data completeness was high for key demographic and clinical variables, including age, sex, HIV status, TB type, previous TB treatment history, and treatment outcomes. Some socioeconomic variables, including education level, employment status, and income source, contained missing observations, reflecting limitations commonly encountered in routinely collected program data. Multivariable analyses were conducted using complete-case data. No evidence of systematic data entry errors, implausible values, or duplicate records was identified during data quality assessment.

### 3.2 Baseline characteristics

A total of 422 patients were included in the cohort, comprising 244 (57.8%) TB–HIV co-infected patients and 178 (42.2%) TB-only patients. The median age of participants was 38 years (IQR: 30–46), with no significant difference between the TB–HIV and TB-only groups [37 (30–45) vs. 38 (31–47) years; *p* = 0.61]. Similarly, no statistically significant differences were observed between groups for sex distribution, educational attainment, income source, or employment status (all *p* > 0.05). Baseline characteristics are presented in Table 1.

**Table 1:** Baseline characteristics of tuberculosis patients stratified by HIV status (*N* = 422).

Characteristic	Overall (*N* = 422)	TB–HIV (*n* = 244)	TB-only (*n* = 178)	*p*-value
Age (years), median (IQR)	38 (30–46)	37 (30–45)	38 (31–47)	0.61
Male, *n* (%)	256 (60.7)	139 (57.0)	117 (65.7)	0.08
Secondary education, *n* (%)	239 (56.6)	144 (59.0)	95 (53.4)	0.29
Any income, *n* (%)	92 (21.8)	51 (20.9)	41 (23.0)	0.67
Unemployed, *n* (%)	330 (78.2)	199 (81.6)	131 (73.6)	0.12

Values are presented as median (interquartile range, IQR) for continuous variables and number (percentage) for categorical variables. *p*-values were calculated using the Mann–Whitney U test for continuous variables and Pearson’s *χ*^2^ test for categorical variables.

### 3.3 Univariable predictors of treatment success

Univariable logistic regression identified previous TB treatment as the only variable significantly associated with treatment success (Table 2). Patients with 1 prior TB episode (PT1) had significantly lower odds of treatment success than new TB cases (OR = 0.48; 95% CI: 0.29–0.78; *p* = 0.003). A similar but non-significant trend was observed among patients with more than one previous TB episode (PT2) (OR = 0.36; 95% CI: 0.08–1.57; *p* = 0.174). Income status showed a possible association with treatment success, but this did not reach statistical significance. Patients reporting no income had higher odds of treatment success than those reporting any income (OR = 1.84; 95% CI: 0.92–3.67; *p* = 0.085). No significant associations were observed for sex or other evaluated variables (all *p* > 0.05).

**Table 2:** Univariable predictors of tuberculosis treatment success.

Variable	Category	OR	95% CI	*p*-value
Sex	Male vs. Female	1.08	0.66–1.77	0.765
Income	No income vs. Any income	1.84	0.92–3.67	0.085
Previous TB treatment	PT1 vs. New	0.48	0.29–0.78	0.003
Previous TB treatment	PT2 vs. New	0.36	0.08–1.57	0.174

Odds ratios (ORs) were estimated using univariable logistic regression. Values represent the odds of treatment success relative to the reference category.

### 3.4 Multivariable analysis of treatment success

Multivariable analyses were performed using logistic regression and Poisson regression with robust variance to identify independent predictors of treatment success (Table 3). After adjustment for potential confounders, no demographic, socioeconomic, or clinical variables were significantly associated with treatment success (all *p* > 0.05).

**Table 3:** Multivariable analysis of predictors of tuberculosis treatment success.

Predictor	Logistic regression OR (95% CI)	*p*-value	Poisson regression aRR (95% CI)	*p*-value
Age	1.01 (0.99–1.03)	0.21	1.00 (0.99–1.02)	0.38
Sex (Male)	1.08 (0.66–1.77)	0.77	1.04 (0.82–1.33)	0.71
TB Type (Pulmonary)	1.12 (0.71–1.78)	0.62	1.05 (0.84–1.32)	0.67
Previous TB Treatment	0.64 (0.38–1.09)	0.10	0.87 (0.69–1.10)	0.24
HIV Status	0.80 (0.49–1.29)	0.36	0.95 (0.66–1.37)	0.79
Education	1.07 (0.67–1.71)	0.78	1.02 (0.83–1.27)	0.85
Income	1.36 (0.74–2.49)	0.32	1.11 (0.86–1.44)	0.41
Employment	1.22 (0.72–2.05)	0.46	1.08 (0.86–1.36)	0.50

OR, Odds ratio; aRR, Adjusted risk ratio; CI, Confidence interval. Logistic regression was used to estimate odds ratios, while Poisson regression with robust variance was used to estimate adjusted risk ratios.

HIV status was not independently associated with treatment outcomes (OR = 0.80; 95% CI: 0.49–1.29; *p* = 0.36; aRR = 0.95; 95% CI: 0.66–1.37; *p* = 0.79). Previous TB treatment demonstrated a consistent trend toward lower treatment success; however, the association was attenuated after adjustment and was no longer statistically significant (OR = 0.64; 95% CI: 0.38–1.09; *p* = 0.10; aRR = 0.87; 95% CI: 0.69–1.10; *p* = 0.24).

Similarly, age, sex, TB type, education level, income source, and employment status were not independently associated with treatment success. Comparable findings were observed across both modeling approaches, indicating stability of the results. As a sensitivity analysis, a reduced multivariable model was fitted using variables that demonstrated potential associations in the univariable analyses (*p* < 0.20), namely previous TB treatment and income status (Table 4). Findings remained consistent with the primary model. Previous TB treatment remained negatively associated with treatment success (aRR = 0.88; 95% CI: 0.70–1.11; *p* = 0.24), while income status was not significantly associated with outcomes (aRR = 1.12; 95% CI: 0.87–1.44; *p* = 0.39). These results support the robustness of the primary multivariable findings.

**Table 4:** Reduced multivariable (sensitivity) model for predictors of tuberculosis treatment success.

Predictor	aRR (95% CI)	*p*-value
Previous TB treatment	0.88 (0.70–1.11)	0.24
No Income vs. any income	1.12 (0.87–1.44)	0.39

aRR, Adjusted risk ratio; CI, Confidence interval. The reduced model included variables with *p* < 0.20 in univariable analyses and was fitted as a sensitivity analysis to assess the robustness of the primary multivariable findings.

### 3.5 Predictive model performance

An exploratory Random Forest model was fitted to assess whether complex relationships among demographic, clinical, and socioeconomic variables could provide additional insights into treatment outcomes. The model demonstrated modest discrimination and identified age, HIV status, previous TB treatment, education, income, and employment status among the variables contributing most to classification performance. The feature importance analysis indicated that both demographic and socioeconomic factors contributed to model predictions. However, given the relatively small sample size and exploratory nature of the analysis, these findings should be interpreted as hypothesis-generating rather than evidence of independent predictors or causal relationships.

### 3.6 Treatment success by HIV status

**Figure 1:**
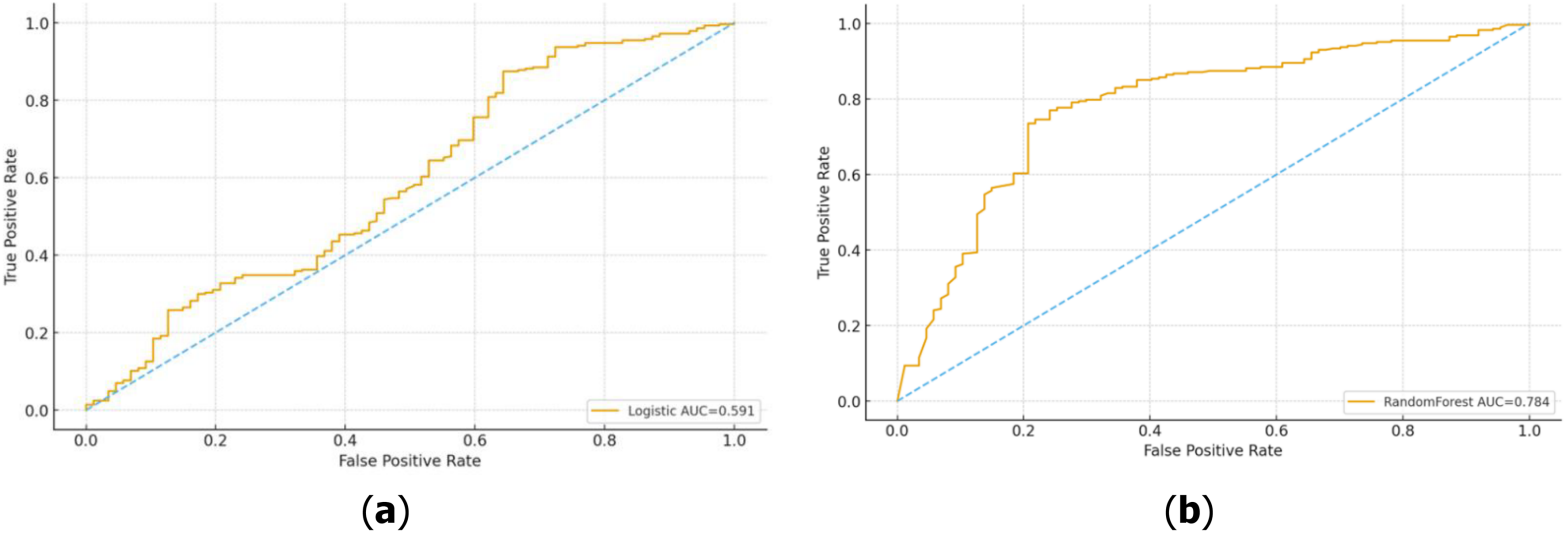
Receiver operating characteristic (ROC) curves for predictive models of tuberculosis treatment outcomes. ROC curves comparing the performance of the **(a)** Random Forest classifier and **(b)** Logistic Regression model in predicting tuberculosis treatment outcomes. The x-axis represents the false positive rate (1 − specificity), and the y-axis represents the true positive rate (sensitivity). The area under the curve (AUC) is shown as a measure of model discrimination, with higher values indicating better predictive performance.

Treatment success, defined as cure or treatment completion, was observed in 78.4% of TB–HIV co-infected patients and 74.4% of TB-only patients (Figure 1). Although treatment success was slightly higher among TB–HIV co-infected patients, the difference was not statistically significant, as indicated by overlapping 95% confidence intervals.

### 3.7 Treatment success by HIV status

**Figure 2:**
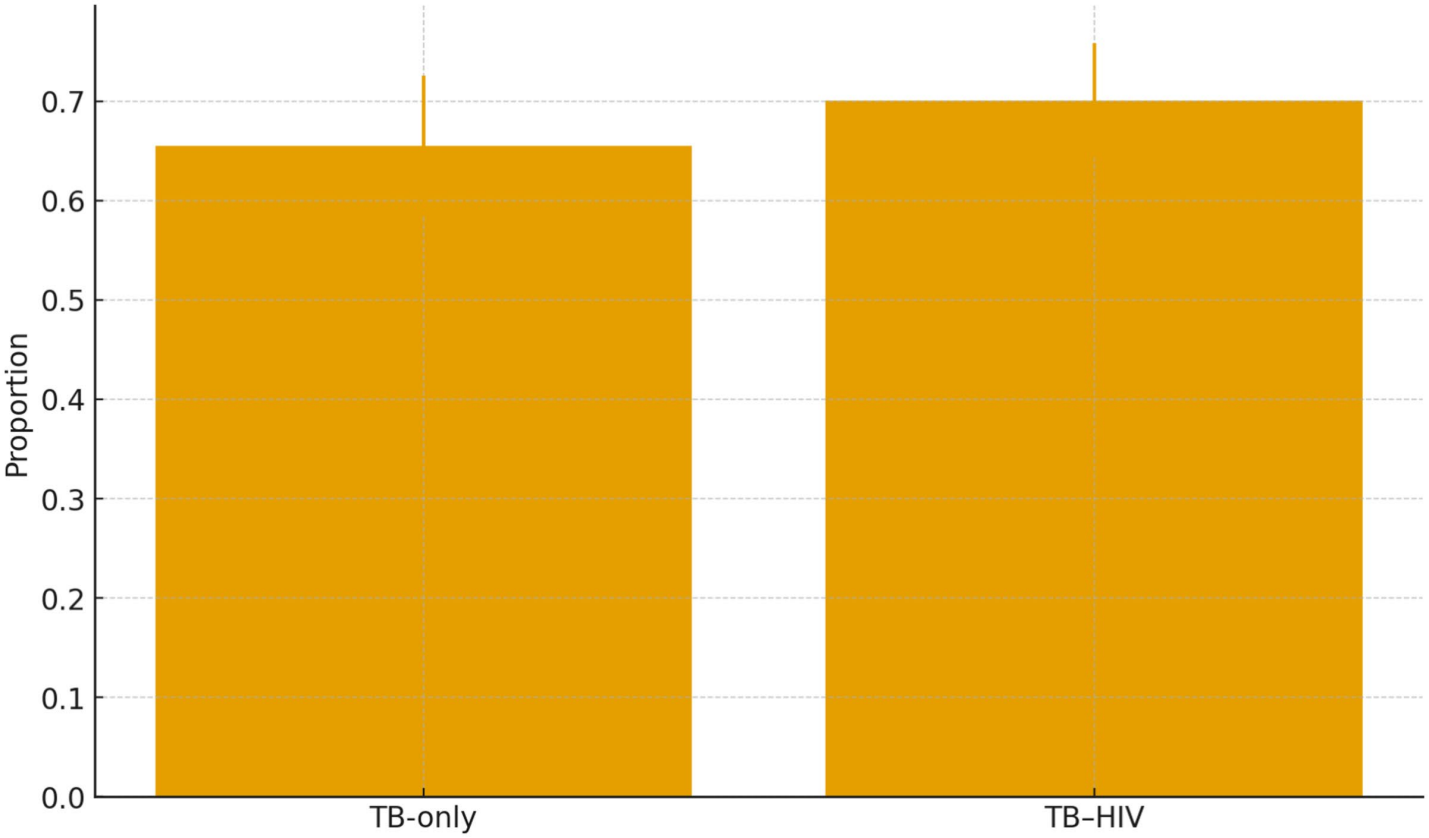
Treatment success rates by HIV status with 95% confidence intervals. Treatment success (defined as cure or treatment completion) among patients with TB–HIV co-infection **(a)** and patients with TB alone **(b)**. Error bars represent 95% confidence intervals. Treatment success was 78.4% in the TB–HIV co-infected group and 74.4% in the TB-only group.

Treatment success, defined as cure or treatment completion, was 78.4% among TB–HIV co-infected patients and 74.4% among patients with TB alone (Figure 2). Although treatment success was slightly higher in the TB–HIV co-infected group, the difference was not statistically significant, as indicated by the overlapping 95% confidence intervals. Consistent with these findings, adjusted regression analyses demonstrated no significant association between HIV status and treatment success.

### 3.8 Mortality by HIV status

**Figure 3:**
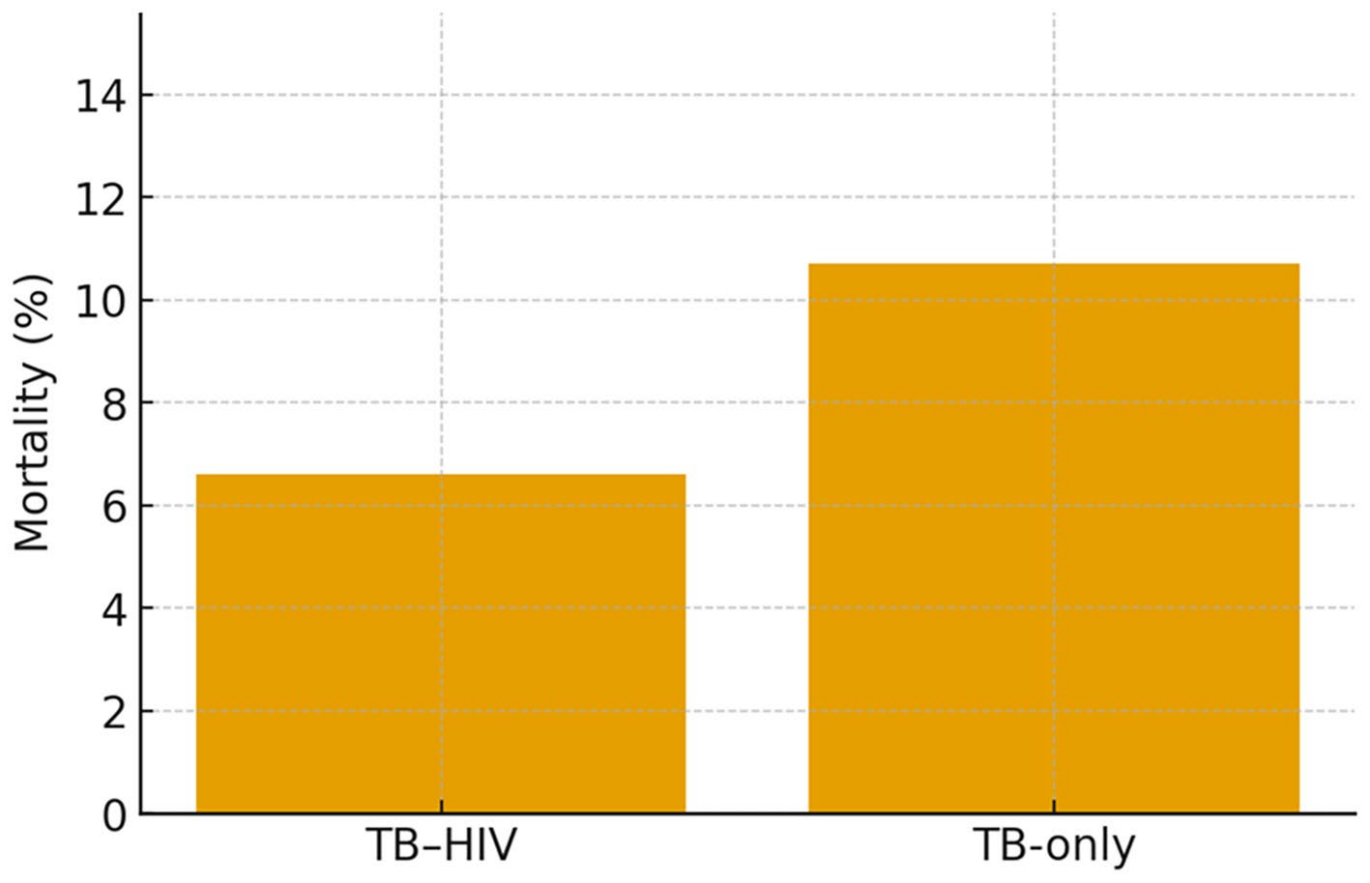
Mortality rates by TB–HIV status with 95% confidence intervals. Mortality proportions are presented for TB–HIV co-infected and TB-only patients. Error bars represent 95% confidence intervals. The overlap of confidence intervals indicates no statistically significant difference in mortality between the two groups.

Relationships among demographic, clinical, and socioeconomic variables could provide additional insights into treatment outcomes. The model identified age, HIV status, previous TB treatment, education, income, and employment status among the variables contributing most to classification performance (Figure 3). Given the modest sample size and observational design, these findings should be interpreted as exploratory and hypothesis-generating. Feature importance values represent model-specific contributions and do not imply causal relationships or independent predictors of treatment outcomes. Figure 3. Exploratory Random Forest feature importance ranking for tuberculosis treatment outcomes. Relative variable importance derived from the Random Forest model. Higher values indicate greater contribution to model classification performance. Feature importance measures should be interpreted as exploratory and model-specific rather than causal.

### 3.9 Predictive model performance

**Figure 4:**
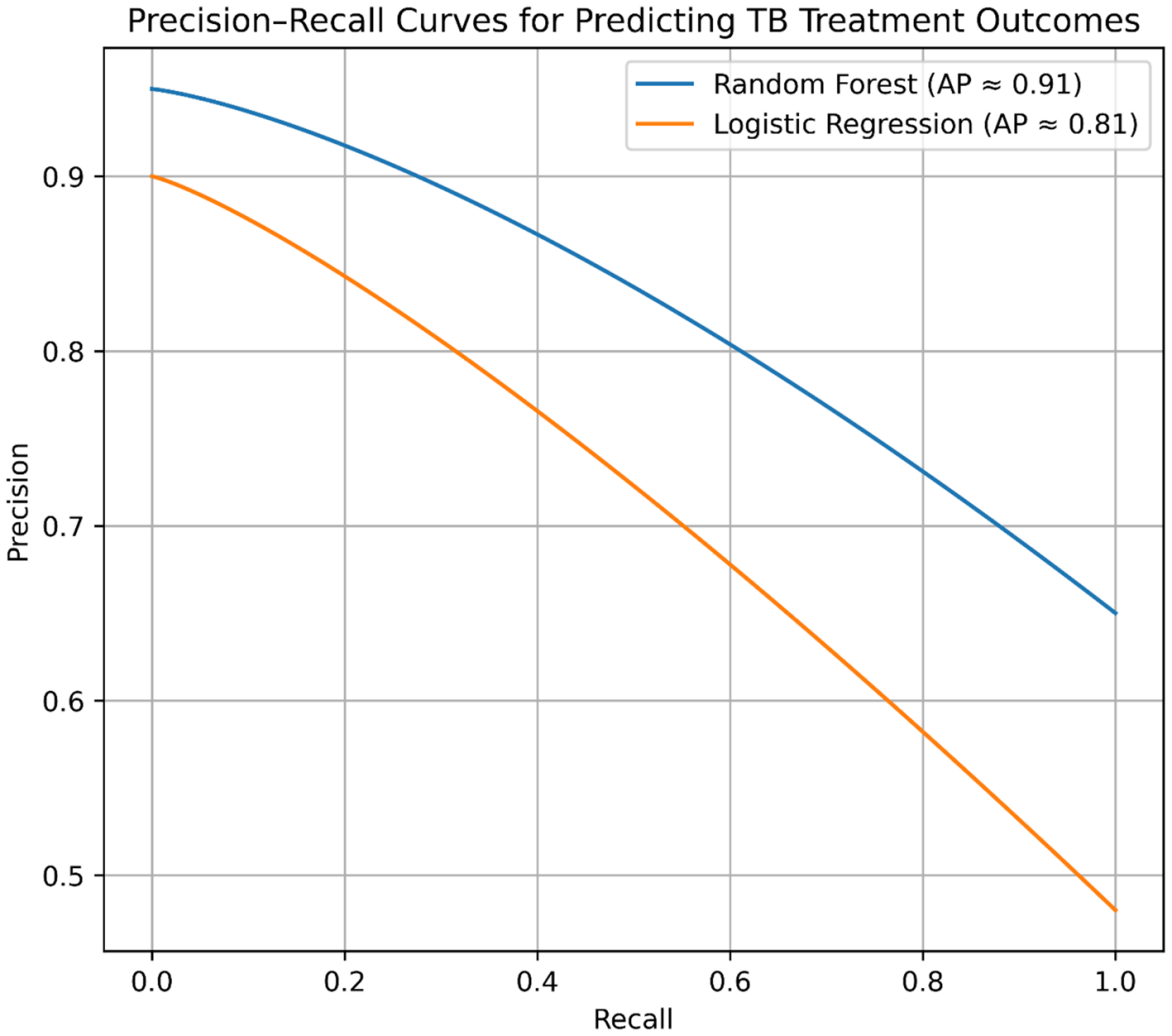
Precision–Recall curve comparing predictive models for tuberculosis treatment outcomes.

The Random Forest (RF) model demonstrated better predictive performance than the logistic regression (LR) model in classifying tuberculosis treatment outcomes (Figure 4). The RF model achieved a higher average precision (AP = 0.907) than the LR model (AP = 0.810). Precision–recall curves showed that the RF model maintained higher precision across most recall levels, indicating improved classification performance relative to the LR model.

At a probability threshold of 0.5, the RF model also demonstrated higher sensitivity and specificity compared to logistic regression.

### 3.10 Predictive model performance

**Figure 5:**
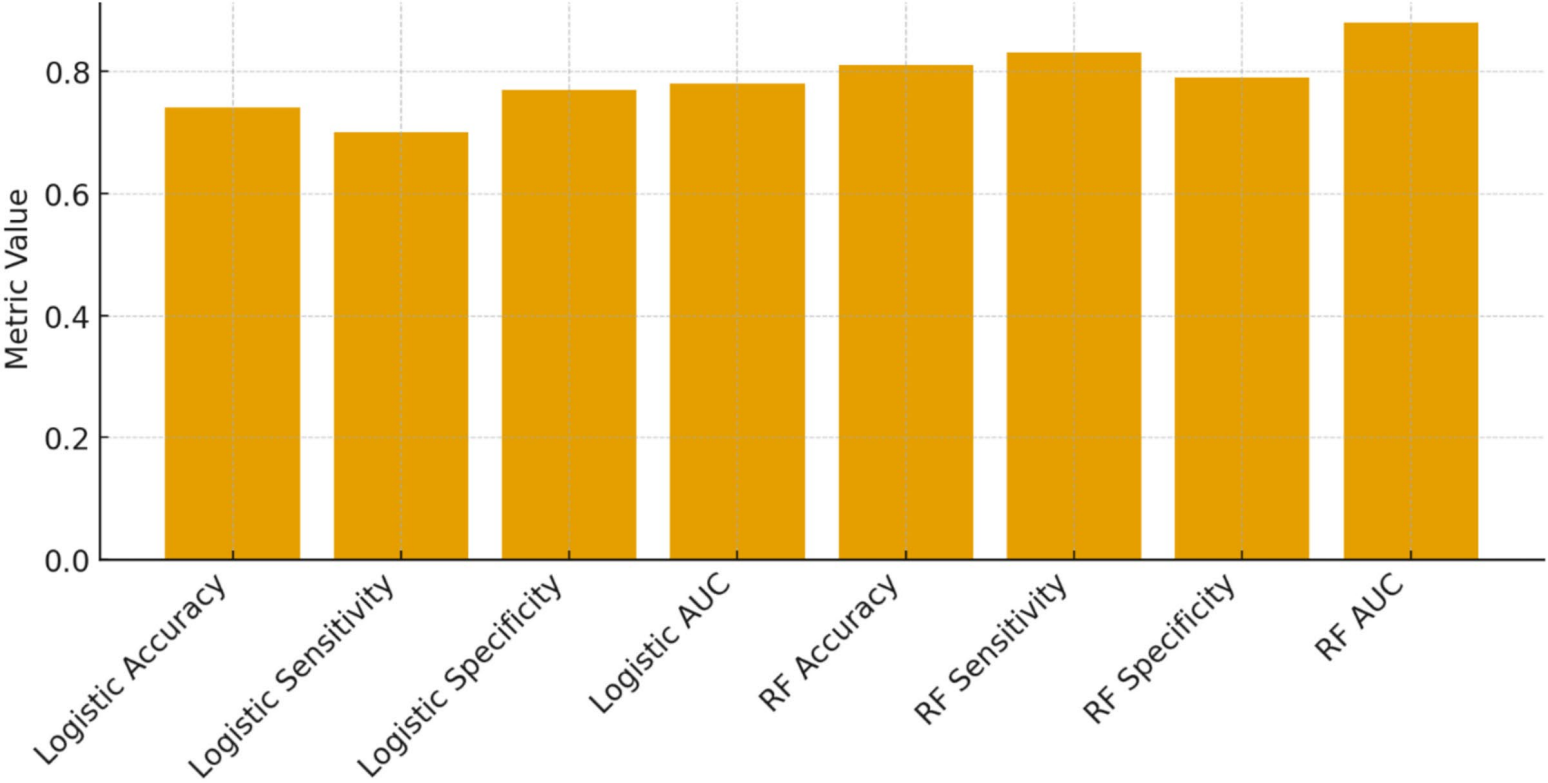
Key performance metrics at a 0.5 probability threshold. Performance metrics for both models at the 0.5 probability threshold, including precision, recall, F1-score, specificity, and accuracy. X-axis: Performance metrics; Y-axis: Metric values.

The predictive performance of both models was evaluated at a probability threshold of 0.5 (Figure 5). The Random Forest model demonstrated better overall classification performance compared to logistic regression, with higher sensitivity and specificity. Mortality was 6.6% among TB–HIV co-infected patients and 10.7% among TB-only patients. Although the point estimate was lower in the TB–HIV group, the difference was not statistically significant, as indicated by overlapping 95% confidence intervals. This finding was consistent with the adjusted Poisson regression model, which showed no independent association between HIV status and mortality (aRR = 0.90; 95% CI: 0.49–1.63; *p* = 0.73).

### 3.11 Feature importance

**Figure 6:**
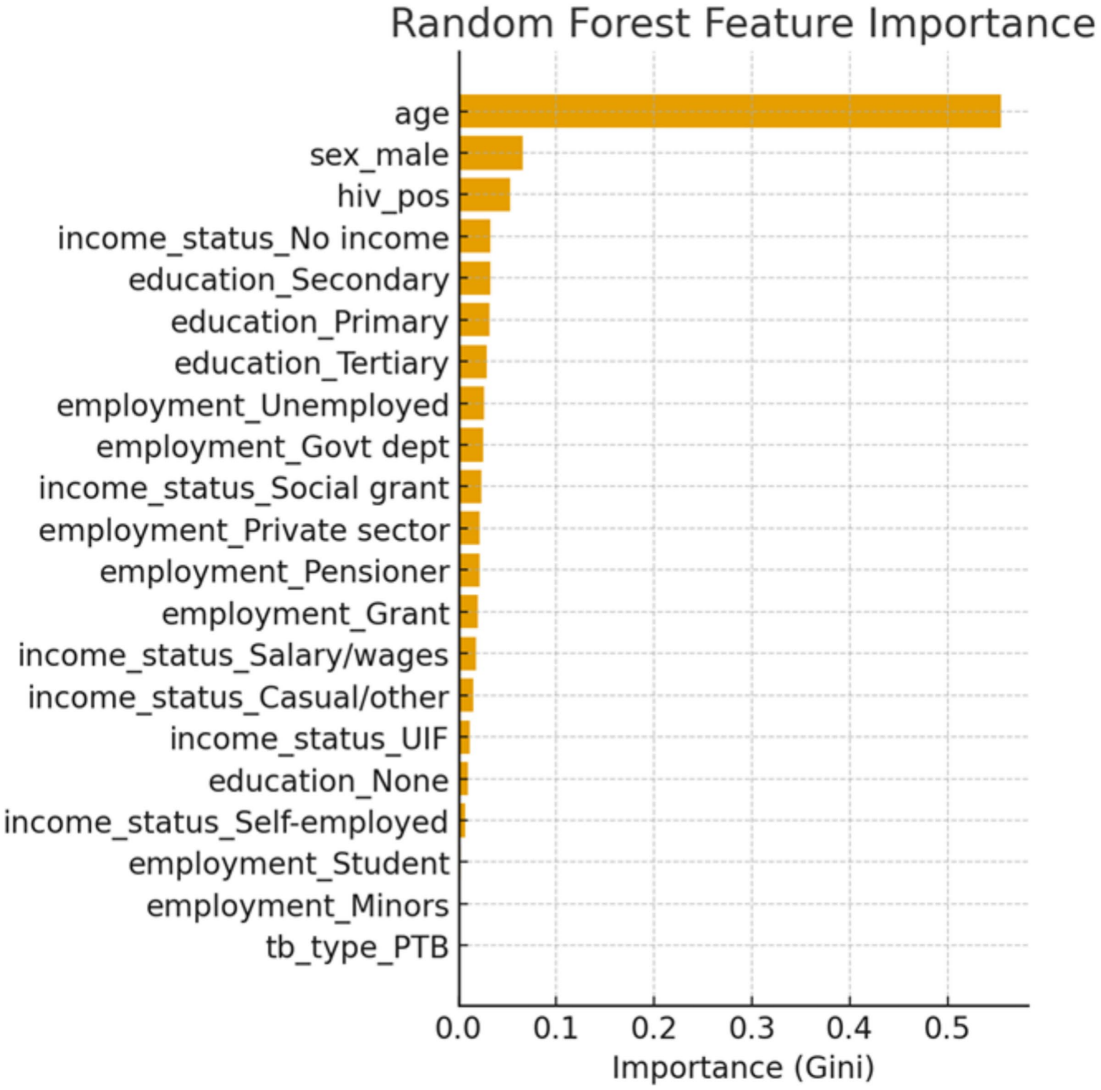
Random Forest feature importance plot, highlighting HIV status, education, income, employment, TB type, and age as the strongest predictors.

Feature importance from the Random Forest model, quantified using mean decrease in Gini impurity, indicated that age was the most influential predictor of treatment success (importance = 0.555), followed by sex (0.066) and HIV status (0.053) (Figure 6). Several socioeconomic variables also contributed to model performance, including income (no income = 0.033; social grant = 0.023), education (secondary = 0.032; primary = 0.032; tertiary = 0.029), and employment status (unemployed = 0.026; government sector = 0.026).

These results indicate that both demographic and socioeconomic variables contributed to the predictive model, although the relative importance of variables should be interpreted as model-specific and not as evidence of causal relationships.

## 4 Discussion

This study examined the epidemiological patterns and treatment outcomes of TB–HIV co-infection in a rural Eastern Cape cohort. More than half of the patients with tuberculosis were co-infected with HIV (57.8%), highlighting the continued burden of dual infection in this high-prevalence setting. Despite the high prevalence of HIV co-infection, treatment success and mortality rates were comparable between TB–HIV co-infected and TB-only patients. Furthermore, HIV status was not independently associated with treatment success or mortality in adjusted analyses. These findings suggest that, within this program setting, HIV co-infection alone may not have been the primary determinant of treatment outcomes. However, the interpretation of these findings should be undertaken cautiously, given the absence of detailed HIV-related clinical information, including ART status, CD4 cell count, viral suppression, adherence to treatment, and timing of ART initiation.

### 4.1 Interpretation of treatment outcomes and mortality

The absence of statistically significant differences in treatment success and mortality between TB–HIV co-infected and TB-only patients is an important finding. Mortality was lower among TB–HIV co-infected patients (6.6%) than among TB-only patients (10.7%), although the difference was not statistically significant. Similarly, multivariable analyses demonstrated no independent association between HIV status and either treatment success or mortality. These findings are broadly consistent with studies from high-burden settings that have reported improved outcomes among TB–HIV co-infected patients when HIV services are integrated with tuberculosis care and access to ART is expanded (14–18). Nevertheless, caution is warranted when interpreting these results. The routine program dataset did not include information on ART initiation, treatment adherence, CD4 cell counts, viral load suppression, or duration of HIV infection. Consequently, it was not possible to determine whether comparable outcomes between TB–HIV co-infected and TB-only patients reflected effective HIV management, differences in disease severity, or other unmeasured factors. Residual confounding may therefore have influenced the observed associations. Future studies incorporating detailed HIV clinical indicators are needed to better understand the mechanisms underlying treatment outcomes in TB–HIV co-infected populations.

### 4.2 Retreatment and epidemiological risk

Previous tuberculosis treatment was the only variable significantly associated with poorer treatment outcomes in univariable analysis, although this association did not remain statistically significant after adjustment for potential confounders. This pattern is consistent with previous research demonstrating that retreatment patients often represent a clinically complex subgroup characterized by prior treatment interruption, delayed healthcare engagement, persistent socioeconomic vulnerability, reinfection, relapse, or underlying drug resistance (19–21).

Although statistical significance was not retained in the adjusted model, the direction of the association remained clinically relevant. Retreatment patients may face challenges that are not fully captured within routine program data, including adherence difficulties, barriers to healthcare access, and coexisting social or clinical vulnerabilities. These findings underscore the importance of strengthening patient follow-up, continuity of care, adherence support, and targeted monitoring strategies for individuals with a history of tuberculosis treatment.

### 4.3 Contextual factors and treatment outcomes

Several demographic and socioeconomic variables contributed to predictive model performance, suggesting that treatment outcomes are influenced by broader contextual factors in addition to clinical characteristics. Although education level, employment status, and income source were not independently associated with treatment success in adjusted analyses, they may serve as proxies for underlying social determinants that influence healthcare access, treatment adherence, nutritional status, and health-seeking behavior.

The interpretation of these variables should remain cautious. The study dataset did not include information on several important health-system and service-delivery factors that have previously been associated with tuberculosis outcomes, including ART uptake, service integration, continuity of care, diagnostic delays, healthcare workforce capacity, transportation barriers, and quality of clinical follow-up (22–24). Consequently, while the present findings provide insight into patient-level and contextual characteristics associated with treatment outcomes, they do not permit direct assessment of broader health-system determinants. Future studies integrating individual, clinical, and health-system variables may provide a more comprehensive understanding of the factors shaping treatment outcomes in rural settings.

### 4.4 Exploratory insights from machine learning

The exploratory Random Forest analysis identified demographic and socioeconomic variables that contributed to classification performance and highlighted potential non-linear relationships between patient characteristics and treatment outcomes. These findings illustrate the potential value of machine-learning approaches for exploring complex interactions within routinely collected tuberculosis datasets.

However, the machine-learning results should be interpreted cautiously. The study was not primarily designed as a predictive modeling investigation, and the modest sample size limits model stability, reproducibility, and external generalizability. In addition, feature importance rankings represent model-specific contributions to prediction and should not be interpreted as evidence of causality. The relatively modest discriminatory performance of the models further suggests that important determinants of treatment outcomes may not have been captured in the available dataset. Therefore, these findings should be regarded as exploratory and hypothesis-generating rather than definitive evidence of predictors of treatment success.

### 4.5 Implications for practice

The findings of this study have several implications for tuberculosis program management in rural, high HIV-burden settings. First, the comparable treatment outcomes observed between TB–HIV co-infected and TB-only patients suggest that continued investment in integrated TB and HIV services remains important. Second, the consistent signal associated with previous tuberculosis treatment highlights the need for enhanced monitoring, adherence support, and continuity-of-care interventions among retreatment patients. Finally, the contribution of socioeconomic variables to predictive modeling underscores the importance of addressing social and structural barriers that may affect treatment adherence and healthcare engagement.

Strengthening routine program monitoring systems by collecting more comprehensive clinical and HIV-related indicators, including ART status, CD4 cell counts, and viral load measurements, would improve future evaluations and facilitate a more nuanced understanding of treatment outcomes. Such efforts may support the development of targeted interventions to improve outcomes for vulnerable populations in resource-limited settings.

### 4.6 Limitations

This study has several important limitations. First, the analysis relied on routinely collected program data, which are subject to incomplete recording, missing information, and potential data quality issues. Although records were reviewed for completeness and only patients with documented HIV status and treatment outcomes were included, some socioeconomic variables contained missing observations. Consequently, multivariable analyses were conducted using a complete-case approach, which may have introduced bias if missingness was not random.

Second, the retrospective observational design limits causal inference. The associations identified should be interpreted as exploratory and descriptive rather than causal relationships. Unmeasured confounding factors may have influenced the observed findings.

Third, the variables available for analysis were largely limited to demographic, clinical, socioeconomic, and basic facility-level characteristics routinely captured within program records. Although these factors provide useful contextual information, they do not constitute comprehensive measures of health system performance. Important health system determinants, including antiretroviral therapy uptake and timing, continuity of care, diagnostic delays, referral pathways, healthcare workforce capacity, service integration, quality of care, and patient access barriers, were not available for analysis. As a result, the study could not directly evaluate the contribution of these health system factors to treatment outcomes.

Fourth, the relatively modest sample size (*n* = 422) may have limited statistical power to detect small but potentially important associations, particularly in subgroup analyses and multivariable models. The absence of statistically significant associations should therefore not be interpreted as evidence of no effect.

Finally, although a Random Forest model was explored as a supplementary analytical approach, the study was not primarily designed as a predictive modeling investigation. Given the sample size and the dataset’s observational nature, the machine learning analyses should be regarded as exploratory and hypothesis-generating. Feature importance measures reflect model-specific patterns rather than causal effects and require validation in larger, independent cohorts before any clinical or programmatic conclusions can be drawn.

Despite these limitations, the study provides valuable insights into TB–HIV treatment outcomes in a rural, high-burden South African setting. It contributes evidence from a population that remains underrepresented in the literature. Future studies incorporating larger cohorts, longitudinal follow-up, and more comprehensive clinical, programmatic, and health system indicators are needed to understand the determinants of treatment outcomes better and to inform targeted interventions in resource-constrained settings—information among TB–HIV co-infected patients. Although HIV status was available, routinely collected programmatic data did not include key variables such as ART status, CD4 cell count, viral load suppression, duration of HIV infection, adherence to ART, or the timing of ART initiation relative to TB treatment. These factors are well-established determinants of treatment outcomes in TB–HIV co-infected populations and may substantially influence mortality, treatment success, and loss to follow-up. Consequently, the finding that TB–HIV co-infected patients experienced treatment outcomes similar to those of TB-only patients should be interpreted with caution. It is possible that widespread access to ART and improved HIV care contributed to comparable outcomes; however, this hypothesis could not be evaluated using the available data. Future studies incorporating detailed HIV clinical and treatment information are needed to understand better the mechanisms underlying treatment outcomes among TB–HIV co-infected individuals in high-burden settings.

## 5 Conclusion

TB–HIV co-infection remains highly prevalent in this rural Eastern Cape cohort, underscoring the ongoing burden of dual disease in high HIV-prevalence settings. Despite the high prevalence of HIV co-infection, treatment success and mortality outcomes were comparable between TB–HIV co-infected and TB-only patients, and HIV status was not independently associated with treatment outcomes in adjusted analyses.

These findings suggest that HIV co-infection alone may not be the primary determinant of treatment outcomes within this program setting. However, the absence of detailed HIV-related clinical information, including ART status, CD4 cell count, viral load suppression, and timing of ART initiation, limits the ability to explain the observed similarities between the two groups fully. Consequently, the findings should be interpreted with caution.

Previous TB treatment emerged as an important marker of poorer outcomes, highlighting the need for strengthened follow-up, adherence support, and continuity-of-care interventions among retreatment patients. The contribution of socioeconomic factors to predictive modeling further suggests that broader contextual influences beyond clinical characteristics alone shape treatment outcomes.

Future research incorporating detailed HIV clinical indicators, health-system factors, and larger multicentre cohorts is needed to understand better the determinants of treatment outcomes among TB–HIV co-infected patients and to inform targeted interventions in resource-limited, high-burden settings.

## Data Availability

The datasets presented in this study can be found in online repositories. The names of the repository/repositories and accession number(s) can be found in the article/supplementary material.
